# Studies on the Aptasensor Miniaturization for Electrochemical Detection of Lead Ions

**DOI:** 10.3390/bios14020110

**Published:** 2024-02-19

**Authors:** Marta Jarczewska, Marta Sokal, Marcin Olszewski, Elzbieta Malinowska

**Affiliations:** 1 Chair of Medical Biotechnology, Faculty of Chemistry, Warsaw University of Technology, Noakowskiego 3, 00-664 Warsaw, Poland; 2 Chair of Drug and Cosmetics Biotechnology, Faculty of Chemistry, Warsaw University of Technology, Koszykowa 75, 00-664 Warsaw, Poland; marcin.olszewski@pw.edu.pl; 3 Center for Advanced Materials and Technologies CEZAMAT, Warsaw University of Technology, 19 Poleczki, 02-822 Warsaw, Poland

**Keywords:** miniaturized electrodes, aptamers, redox indicators, voltammetry

## Abstract

Lead poses severe effects on living organisms, and since Pb^2+^ ions tend to accumulate in different organs, it is crucial to monitor Pb^2+^ concentration in samples such as water and soil. One of the approaches is the utilization of biosensors combined with aptamer-based layers for the electrochemical detection of lead ions. Herein, we present the studies of applying miniaturized screen-printed transducers as solid surfaces to fabricate aptamer layers. As the research is the direct continuation of our previous studies regarding the use of gold disk electrodes, the working parameters of elaborated aptasensors were defined, including the range of linear response (10–100 nM), selectivity as well as stability, regeneration, and feasibility of application for the analysis of real samples. This was achieved using voltammetric techniques including cyclic and square-wave voltammetry in the presence of methylene blue redox indicator.

## 1. Introduction

Monitoring ground and surface water quality is crucial because drinking water sources are constantly exposed to contamination caused by the development of areas such as mining, agriculture, or urbanization. One of the most dangerous pollutants is heavy metals, including lead. In the past, lead was applied for the fabrication of pipes or pottery, and it is still used to produce car batteries, pigments, ammunition, roofing, and stained-glass windows in architecture [1]. Lead causes severe effects on human health since it can accumulate in various organs, resulting in damage to muscles, the renal system, and the central nervous system [2]. The latter one, affected by lead poisoning, is especially harmful to young children in terms of the possibility of disorders, such as reduction of neurocognitive potential and increased aggressiveness [2]. It was stated by the Centers for Disease Control and Prevention (USA) that lead concentrations below the level of concern are currently below 10 µg/dL. In contrast, very high levels are defined as above 70 µg/dL [3] and can result in encephalopathy, coma, or even death [4]. This highlights the need to elaborate novel methods for Pb^2+^ ions detection characterized by high sensitivity and selectivity. Conventionally, lead ions detection techniques such as capillary electrophoresis, atomic absorbance spectrometry [5,6], and inductively coupled plasma spectrometry [7] were utilized. The main disadvantage of such methods is the need for sophisticated equipment and a trained operator. To further cope with the challenge of limited portability and the necessity of sample pretreatment, which are the obstacles of contemporary applied methods for lead detection, electrochemical methods were utilized, including potentiometry combined with ion-selective electrodes (ISE) [8] using ionophores [9] as well as voltammetry [10,11,12]. In the past, lead ions were detected with high sensitivity using polarography [13,14] and stripping techniques such as differential-pulse anodic stripping voltammetry (DPASV) [15], square-wave anodic stripping voltammetry (SWASV) [16], and potentiometric stripping analysis [17], reaching detection limits of 31 pM [18]. Recently, several approaches to Pb^2+^ ions detection using chemical receptors and nanomaterials were tested, providing nanomolar or picomolar detection limits, including the application of kaolinite [19], graphene/bismuth nanocomposite [20], or carbon-covered halloysite [21].

**Scheme 1:**
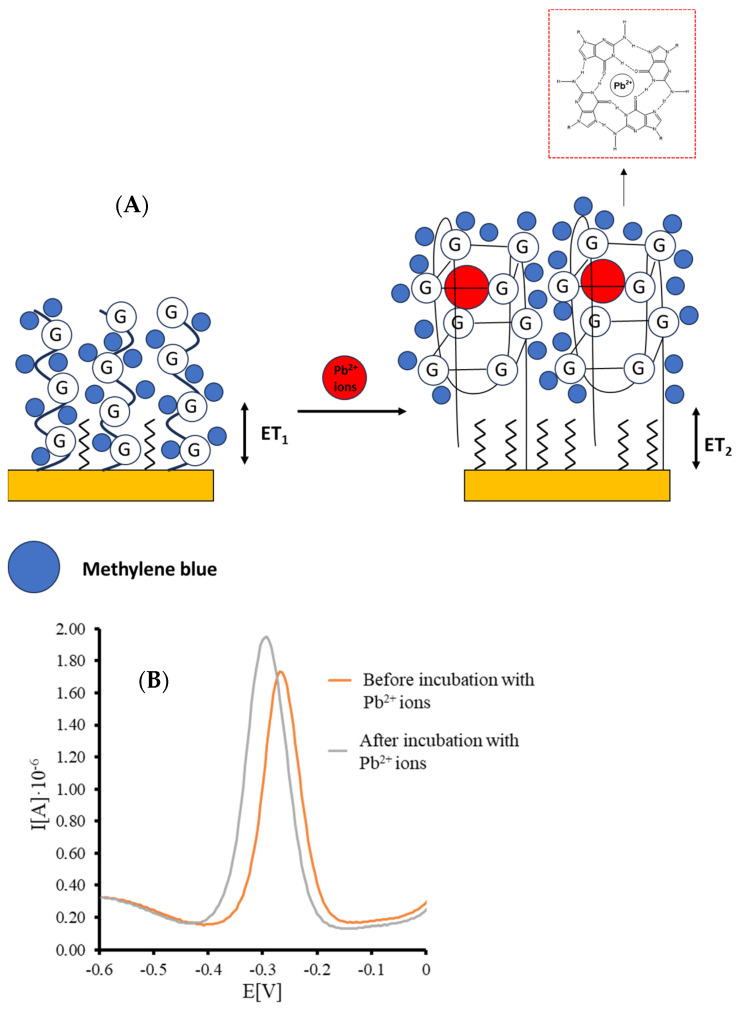
(**A**) Representation of TBA/MCH-based receptor layer upon interaction with lead ions in the presence of methylene blue redox indicator, including the formation of G-quadruplex structure. (**B**) An example of the square-wave voltammograms (anodic scan) recorded for aptamer/MCH-modified miniaturized electrode before and after incubation with a solution containing Pb^2+^ ions.

During the last few years, the possibility of introducing nucleic acids as receptor layers of biosensors dedicated to detecting lead ions has been widely studied. Random single-stranded DNA or RNA might not maintain sufficient selectivity towards lead ions due to sugar–phosphate moieties that attract several positively charged species, including metal cations. In such cases, functional nucleic acids such as DNAzymes [22,23] and aptamers [24,25] were employed as receptor elements [25,26,27,28,29,30,31]. Usually, DNAzymes that contain a specific region for lead ion binding require the presence of a substrate sequence so that they can form a duplex structure. After the introduction of a target analyte, a substrate strand becomes cleaved, releasing its fragments. A substrate structure is often labeled with fluorophore and quencher molecules that generate an optical signal upon separation of substrate sequence pieces from the hybridized form. The main disadvantages of the proposed system are the relatively complex recognition process and the limitation of optical detection techniques in terms of sample characteristics like homogeneity. On the contrary, applying aptamers enables the utilization of several detection techniques, such as electrochemical, mass, and optical. Aptamers are short, single-stranded DNA or RNA molecules that change their conformation upon interaction with target analytes such as metal cations, proteins, and cells [32,33]. So far, aptamers have been used in analytical methods such as affinity chromatography, as drugs (e.g., Macugen), and as receptor layers in biosensors [33]. The latter application can be achieved as aptamers can be easily conjugated with groups, allowing surface immobilization. Some groups, such as hydroxyl (2′-OH) in the ribose ring, can be substituted, for instance, with 2′-F [33], to enhance sequence stability in the presence of nucleases. Moreover, introducing an electroactive or fluorescent label at one of the terminal ends of the sequence provides a source of analytical signal. It should be emphasized that even though most of the aptamers were identified for high molecular weight targets, there are examples of sequences of high affinity towards metal cations, including potassium [34], cadmium [35], copper [36], and lead [37]. An electrochemical aptasensor dedicated to detecting potassium ions was the first example of applying an aptamer-based layer for the analysis of metal cations [38]. In such an approach, thrombin binding aptamer (TBA) was immobilized on a gold disk electrode surface, and a ferrocene label was conjugated to its 5′ end. A G-quadruplex structure was formed upon the addition of potassium cations, leading to a limitation of the distance between the redox indicator and the electrode surface. Consequently, the current response increased after electrode incubation with K^+^ ions. Such a system known as electronic-beacon assay (e-beacon) was also developed for the detection of other targets, including theophylline [39], PDGF [40], or VEGF protein [41]. However, it should be noted that using the e-beacon construct might result in a current response of low sensitivity compared to the case where label-free sequences are utilized for surface functionalization and soluble electroactive indicators are the source of the current response. Interestingly, it was shown that potassium ions do not solely cause the rearrangement of G-rich sequences into G-quadruplexes; they can also be formed when Pb^2+^ is present. Hence, for the detection of lead ions, aptamers abundant in guanine moieties were mainly applied, with some examples including the conjugation of guanine-rich sequences with fluorescent labels or redox active molecules. Our previous paper employed this approach, which focused on utilizing TBA aptamer [42] as a sensing layer and gold disk electrodes as transducers. In contrast, methylene blue was applied as a redox indicator. The aptamer-based layer switch upon binding to lead ions was confirmed by comparison of impedimetric spectra recorded for gold disk electrode modified with TBA sequence or DNA single-stranded probe of the same length, which did not contain any guanine residues. A decrease in charge transfer resistance was evident in the TBA—the TBA-based layer shown in the Nyquist plot, while for the ssDNA-modified electrode, no change in impedimetric spectra was visible. To provide a high-sensitivity aptasensor, a soluble electroactive molecule—methylene blue—was introduced to generate an analytical signal. Upon interaction of methylene blue with the sensing layer, an initial current response was recorded, followed by aptasensor incubation in a solution containing methylene blue and lead ions. This caused the aptamer strand switch and formation of G-quadruplex stabilized by Pb^2+^ ions. Moreover, as guanine moieties exhibit high affinity towards methylene blue, redox indicator molecule accumulation was observed that increased the current signal (Scheme 1).

**Figure 1:**
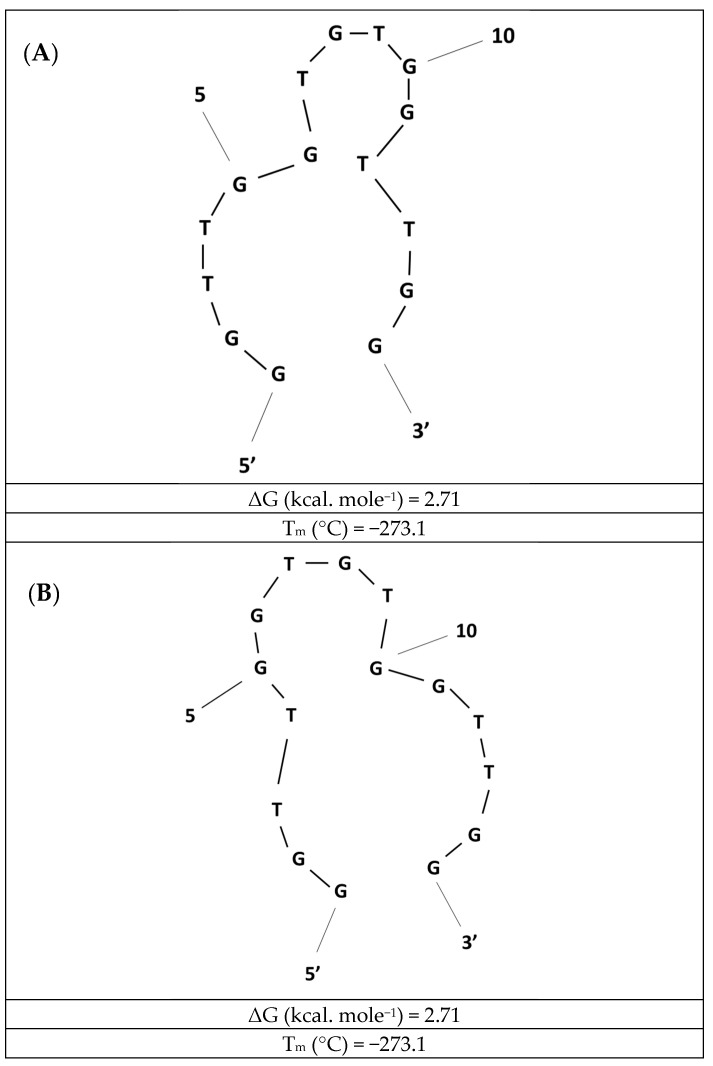
Examples of the two most favorable hairpin structures (**A**,**B**) formed by thrombin-binding aptamer (TBA) in the absence of lead ions that are distinguished with the highest stability before the addition of the target analyte. The Integrated DNA Technologies (IDT) analysis tool obtained the structures, which were redrawn.

In most approaches, thrombin-binding aptamers (TBAs) were expected to form guanine tetrads (G-tetrads) with specific target molecules such as thrombin, potassium, or lead ions. These structures might be present within a single sequence. However, the possibility of G-quadruplex formation based on two or even four guanine-rich sequences cannot be excluded. On the contrary, in the absence of a target analyte, such as lead ions, the initial aptamer conformation might not provide the upright orientation of aptamer strands, as other secondary structures, such as hairpin structures, might also be formed. As shown in Figure 1, such structures contain two hairpins, and such sensing layer conformation might influence the effectiveness of switching it into G-tetrad structures. Concerning the use of thrombin-binding aptamers as receptor layers and their immobilization on solid surfaces, the likelihood of the formation of a defined structure will strongly depend on DNA aptamer surface density. This is particularly crucial in applying miniaturized transducers, distinguished by smaller geometrical areas and less expanded electrochemical working areas. Nevertheless, the mechanism of G-quadruplex formation and subsequent accumulation of methylene blue molecules in close vicinity to the electrode surface is the most probable mechanism of aptamer-based layer arrangement.

Herein, a direct continuation of the studies regarding aptamer-based systems was executed using commercially available screen-printed gold electrodes. The use of such transducers was justified by the aim to minimize the amounts of chemicals required to fabricate sensing layers and perform electrochemical measurements. It should be emphasized that scaling down the biosensor dimensions provides a detection tool that can be utilized “in situ” without the necessity of sample transportation to a specialized laboratory, reducing the analysis time. The aptamer layer formation procedure was recreated concerning the protocol optimized for gold disk microelectrodes [42], along with the conditions for performing voltammetric measurements, including cyclic and square-wave voltammetry application. The experiments enabled the definition of working parameters such as the range of linear response and selectivity and the possibility of analysis of real sample tap water spiked with lead ions. The results indicated the possibility of aptamer-based sensor miniaturization and detection of lead ions in the range (10–100 nM) overlaying the maximum safe limit set by the US EPA (72 nM) [43,44], which can be applied for analysis of waters with upregulated lead level rather than for water samples not characterized with higher lead ions concentration. Such an approach confirms the possibility of using a developed tool for the electrochemical analysis of lead ions, which could be an alternative to contemporary methods.

## 2. Materials and Methods

### 2.1. Apparatus

Electrochemical measurements were conducted using cyclic voltammetry (CV), square-wave voltammetry (SWV), and electrochemical impedance spectroscopy (EIS) with the application of CHI660 potentiostat (CH Instruments, Austin, TX, USA) and PalmSens4 potentiostat (Palmsens, Houten, The Netherlands). The experiments were executed using miniaturized screen-printed electrodes containing a gold working electrode, pseudoreference electrode—Ag covered by AgCl and Au counter electrode at room temperature. Cyclic voltammetry was conducted in the presence of methylene blue redox indicator at a scan rate of 0.1 V·s^−1^ and square-wave voltammetry at a pulse amplitude of 10 mV, increment of 5 mV, and frequency of 15 Hz. A potential range from −0.6 to 0 V was applied for both techniques. For EIS measurements, a potential 0.2 V for the frequency in the range from 1 Hz to 50 kHz was used in the presence of a 5 mM potassium ferri/ferrocyanide redox indicator.

### 2.2. Reagents

Potassium hexacyanoferrate (III) (K_3_Fe(CN)_6_), potassium hexacyanoferrate (II) (K_4_Fe(CN)_6_), methylene blue (MB), Tris-HCl, manganese(II) nitrate hydrate, uranyl acetate, lead nitrate, cadmium nitrate, mercury chloride, sodium selenate, nickel sulfate, potassium antimony(III) tartrate hydrate, arsenic(III) ICP standard, 6-mercapto-1-hexanol (MCH), and potassium dihydrogen phosphate were purchased from Aldrich Chemicals, Darmstadt, Germany. Nitrilotriacetic acid (NTA) was purchased from Alfa Aesar, Haverhill, MA, USA. 

DNA aptamer probe (desalted, purified by HPLC) specific for lead ions and reference ssDNA sequences were purchased from Metabion, Planegg, Germany. The aptamer sequence was 5′-OH-C_6_-S-S-C_6_-GGT TGG TGT GGT TGG-3′ and the reference one was 3′-OH-C_3_-S-S-C_3_-TTT TTT TTT TTT TTT-5′. Aptamer stock solutions (100 μM) were prepared with nuclease-free water and stored in a −20 °C freezer before use.

### 2.3. Solutions

The solutions used in the experiments were as follows: 50 mM Tris-HCl, pH 4.0, 5 mM ferri/ferrocyanide in 0.1 M KCl, 50 µM MB in 50 mM Tris-HCl, 1 M KH_2_PO_4_, pH 4.5, 4 μM MCH in 1 M KH_2_PO_4_. Methylene blue solutions were spiked with lead nitrate or other heavy metal salts if necessary.

### 2.4. Gold Electrode Cleaning and Modification

As gold transducers are very fragile, applying strong acids and oxidizers such as piranha solution for surface cleaning was impossible. The working electrode surface cleaning was limited to washing with water and ethanol, which was followed by verification of the purity of the gold surface using cyclic voltammetry in 5 mM ferri/ferrocyanide in 0.1 M KCl (scan rate 0.1 V·s^−1^, potential range from −0.2 to 0.6 V, 2 cycles). The electrodes were immediately modified by 2 h incubation with 4 μM aptamer (in 1 M KH_2_PO_4_), followed by 1 h incubation with 4 μM MCH blocking agent.

## 3. Results and Discussion

Our previous studies revealed the possibility of applying thrombin-binding aptamers (TBA) as receptor layers for detecting lead ions combined with gold disk electrodes as transducer elements [42]. In this case, we observed a current increase for TBA-modified gold macroelectrodes upon incubation with lead ions in the presence of MB redox indicator compared to the current recorded by incubation with solely methylene blue. This indicated a substantial change in TBA-based layer conformation and the occurrence of G-quadruplex structure formation stabilized by lead ions. It should be noted that a G-quadruplex structure might be formed not only within a single strand but also by two or even four oligonucleotide strands. Nevertheless, a G-quadruplex structure exposes guanine moieties in the outer part of the receptor layer. As a result, the access for methylene blue towards guanine moieties is enhanced, and as it was previously shown, methylene blue preferentially binds to guanine moieties [45,46].

The binding between the metal cation and G-quadruplex is dependent on the ions present at the central channel that is formed because of the quartet arrangement. Ions can be located between the planes of G-quartets or along the quartets. Through molecular dynamics studies [47] it was shown that the G-quadruplex structure is destabilized in the absence of certain cations. Initially, the possibility of the formation of G-quadruplex was evidenced in the presence of potassium or sodium ions using, e.g., circular dichroism [48,49]; however, such structures might be stabilized not only by monovalent ions but also by some divalent ones, including Sr^2+^, Ba^2+^, and Pb^2+^, where the interaction between oxygen and metal is responsible for stabilization [50]. It was stated that numerous monovalent and divalent cations might be engaged in stabilizing the G-quadruplex structure and the following orders were proposed by Hardin: K^+^ > Ca^2+^ > Na^+^ > Mg^2+^ > Li^+^ and K^+^ > Rb^+^ > Cs^+^ [51] and by Venczel: Sr^2+^ > Ba^2+^ > Ca^2 +^ > Mg^2+^ and K^+^ > Rb^+^ > Na^+^ > Li^+^ = Cs^+^ [52]. ESI-MS studies on G-quadruplex formation deoxyguanosine showed that the stability with divalent cations is as follows: Sr^2+^ > Ba^2+^ > Pb^2+^ > Ca^2+^ >> Mg^2+^ and metal–oxygen bond oversees stabilization of G-quadruplex structure [53]. In some cases, when the temperature is high, or there is a low concentration of monovalent ions, divalent cations, including Mn^2+^, Ni^2+,^ or Mg^2+,^ can cause the destabilization of G-quadruplexes [54]. It should also be noted that the main feature for choosing the cation for G-quadruplex formation is the ionic radius, and small ions such as sodium ions might be coordinated within the plane of G-tetrad in contrast to potassium ions that are between the planes [55].

Pb^2+^ ions are also concerned with preserving G-quadruplex structure [56,57,58], which are formed on a three-step pathway [59]. The affinity of lead ions towards guanine-rich ssDNA strands was shown and utilized to develop biosensors for lead ion detection [25,60,61,62], though optical-based sensors dominated it. Nevertheless, there are also examples of the utilization of electrochemical aptamer-based sensors for lead ions detection [63,64]. Such mechanism was also studied regarding structural details on binding between G-quadruplexes and lead ions. Therefore, a lipophilic guanosine analog was applied, and a complexation with Pb^2+^ ions to yield G-quartets and G8 octamers in solid-state and solution was studied [65]. Interestingly, NMR and crystal structures of G8 complexes indicated that a smaller and highly charged Pb^2+^ divalent ion templates a smaller G8 octamer cage than a potassium ion. This denotes tighter coordination between Pb^2+^ ions, and the G-octamer is more kinetically stable than the G8–K^+^ complex. Therefore, the binding between Pb^2+^ and G-rich single-stranded DNA is characterized with higher stability than observed for potassium–G-rich single-stranded DNA complex. Moreover, the binding between thrombin-binding aptamers and lead ions studied by Extended X-Ray Absorption Fine Structure (EXAFS) happened between two quartets, coordinating with the eight surrounding guanine O6 atoms [66].

Hence, we decided to combine the exceptional feature of G-quadruplex formation upon the presence of lead ions followed by evidencing such interaction using methylene blue as a redox indicator, which showed high affinity towards guanine moieties exposed after G-quadruplex formation using gold macroelectrode transducers [42]. Eventually, we attempted to recreate the aptamer-based layer on the surface of miniaturized three-electrode systems.

After the formation of the sensing layer, a comparison of responses expressed was made:

(1) $$Aptasensor\;response=\frac{\left(I-I_{0}\right)}{I_{0}}$$

where I_0_ was cathodic current recorded in 50 µM methylene blue (in 50 mM Tris-HCl, pH 4.0) before and I recorded in 50 µM methylene blue in 50 mM Tris-HCl, pH 4.0 after electrode incubation with lead ions (in 50 µM methylene blue in 50 mM Tris-HCl, pH 4.0).

**Figure 2:**
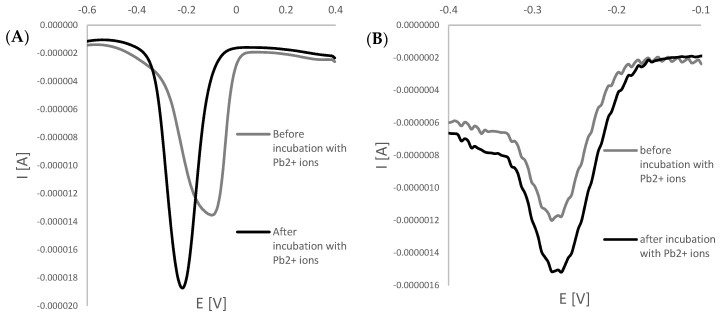
Examples of square-wave voltammograms (cathodic scans) were obtained for macro (**A**) and miniaturized electrodes (**B**) before and after incubation with lead ions.

The equation, as mentioned above, allowed us to minimize the influence of the electrochemical area of each single-use electrode on the average response of the aptasensor at defined conditions. It should be noted that there is a significant difference between gold disk macroelectrode and miniaturized gold electrode area, as the former is defined with 3.14 mm^2^ and the latter with 4 times smaller, reaching 0.785 mm^2^ that results in the intensity of miniaturized electrodes being one magnitude lower than for macroelectrodes. Nevertheless, the tendency of current change upon interaction with lead ions was observed (see Figure 2). Moreover, as aptasensor response derived from SWV cathodic measurements was distinguished with higher sensitivity than anodic one, the square-wave cathodic voltammograms were processed and utilized for defining the working parameters of miniaturized aptamer-based biosensors such as range of linear response, selectivity, regeneration, and stability.

**Figure 3:**
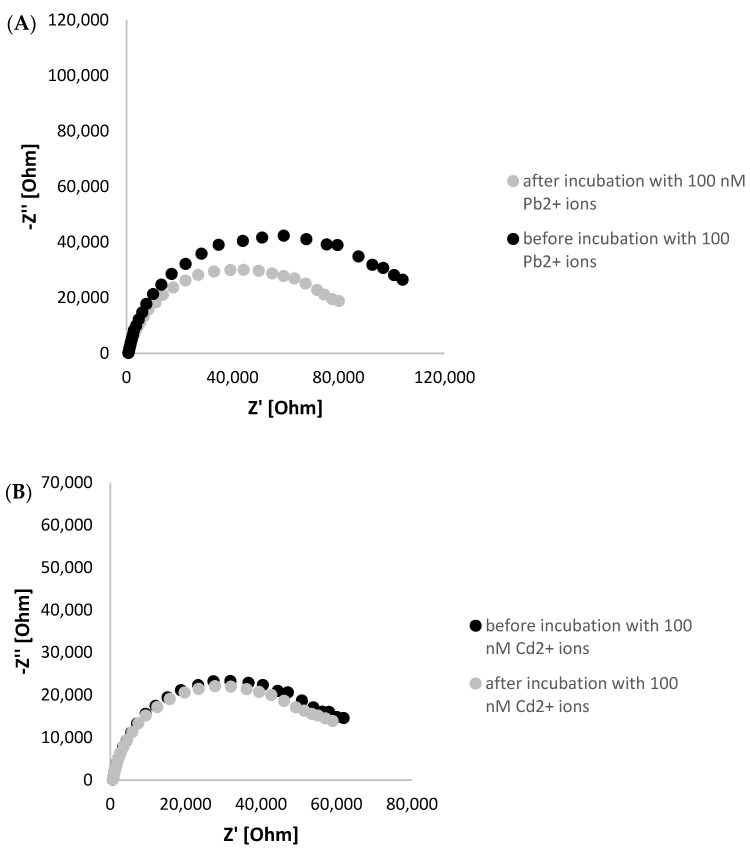

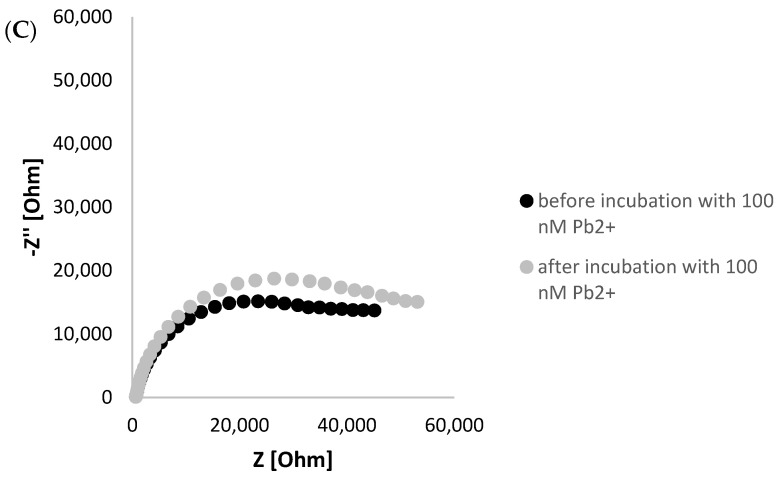
Nyquist plot presenting impedimetric response of TBA/MCH-modified miniaturized electrode before and after incubation with 100 nM lead ions (**A**), 100 nM Cd^2+^ ions (**B**), and polyT/MCH-based layer with 100 nM Pb^2+^ ions (**C**). The EIS studies were conducted in the presence of 5 mM ferri/ferrocyanide redox indicator (in 50 mM Tris-HCl, pH 4.0), and the applied frequency range was from 1 Hz to 50 kHz.

To further confirm the possibility of G-quadruplex formation, electrochemical impedance experiments were executed in the presence of ferri/ferrocyanide redox couple as a source of aptasensor response. As seen in Figure 3A in A Nyquist plot, a R_et_ decrease was observed after incubation of TBA/MCH-based layer with 100 nM lead ions. This evidenced the rearrangement of the G-rich aptamer sequence into a layer of a smaller thickness, leading to a smaller distance between the ferri/ferrocyanide redox indicator that enables more efficient charge transfer between the redox indicator and electrode surface. On the contrary, a minor R_et_ decrease was recorded when TBA/MCH-based miniaturized electrode was incubated with 100 nM Cd^2+^ ions, which might be justified by partial reduction of overall aptamer layer negative charge through binding with cadmium ions (Figure 3B). This might slightly enhance the attraction of ferri/ferrocyanide redox indicator to the receptor layer. Furthermore, a formation of G-quadruplex is unique for guanine-rich sequences as for polyT/MCH-based layer; a slight increase in charge transfer resistance was observed after incubation with 100 nM Pb^2+^ ions, as seen in Figure 3C. This indicates that there are interactions between aptamer strands within the layer, resulting in a steric hindrance for redox indicator and less efficient charge transfer.

**Figure 4:**
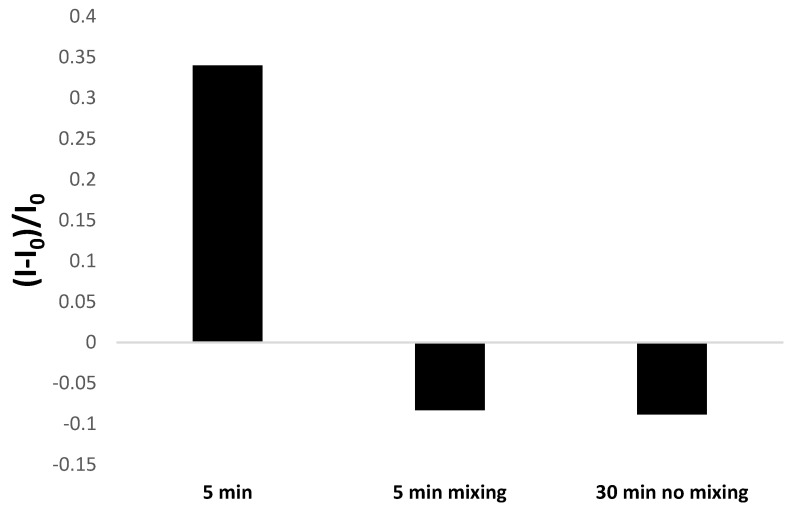
Comparison of the influence of aptamer-based electrode incubation conditions in lead nitrate solution (100 nM Pb(NO_3_)_2_) in 50 mM Tris-HCl, pH 4.0 containing 50 µM methylene blue). The aptasensor response was based on square-wave voltammetry (cathodic scan) results in the presence of 50 µM methylene blue redox indicator.

**Figure 5:**
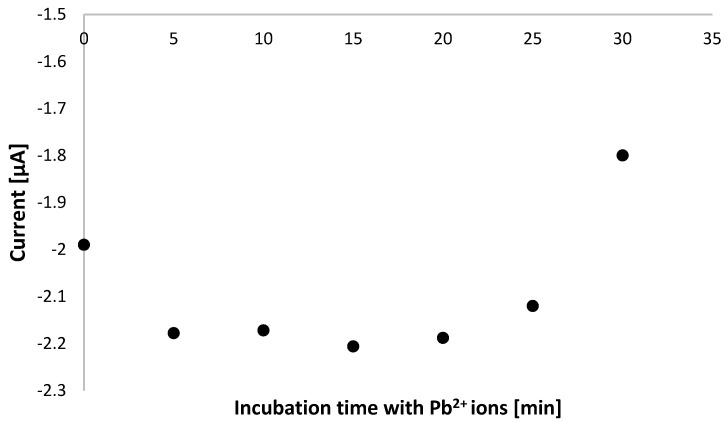
Dependence of cathodic current intensity versus incubation period of TBA/MCH-modified miniaturized electrodes in 100 nM Pb^2+^ solution containing methylene blue redox indicator (in 50 mM Tris-HCl, pH 4.0).

Initially, the conditions for executing voltammetric measurements to evidence the binding between aptamer-based layer with lead cations were selected and referred to incubation time of modified electrode with methylene blue solution spiked with Pb^2+^ ions. As can be seen in Figure 4, there is a substantial difference between responses recorded after 5 min incubation with 100 nM lead ions at static conditions, which reaches 0.35, whereas both at stirring conditions after 5 min and 30 min incubation at static conditions, the response does not exceed −0.1 and evidence a minor current drop after incubation with lead ions. In the case of stirring conditions, this could be justified by the partial deterioration of the aptamer-based layer and the possibility of pinholes in the receptor layer available for the adsorption of methylene blue molecules. Though lead cations interaction with remaining aptamer strands can still happen, the alteration of receptor layer properties results in a negligible current change. When longer than 30 min, incubation time at static conditions was tested, and similar behavior in terms of aptasensor response was evidenced, which can also indicate the possible damage of the aptamer layer upon prolonged contact with the target analyte or, even more likely, a change of the composition of the receptor layer. The latter might be caused by distinct conformations of aptamer strands forming the receptor layer and indicates the dynamics of the aptamer-based layer. The possibility of reorganization from intrastrand to interstrand G-quadruplexes cannot also be excluded. It was shown that Pb^2+^-induced folding of TBA into a G-quadruplex occurs probably through the rapid formation of an intermediate Pb^2+^-TBA complex that then isomerizes to the fully folded structure [67]. This might be why a more pronounced response was recorded after 5 min incubation with lead ions after a 30-min period. Based on such reports, we also aimed to verify the effect on aptamer-based layer incubation time with lead ions, and it can be observed in Figure 5 that a signal increase is visible after subsequent incubations in lead nitrate solution containing MB redox indicator, which eventually decreases after 30 min incubation. This might result from a combination of factors, including various conformations of aptamer strands, their dynamics, and the possibility of a change from intrastrand to interstrand G-quadruplexes. Based on such results, a shorter incubation with lead ions is more favorable regarding the proposed aptasensor’s sensitivity. As 5 min static incubation with lead cations solution provided the most significant response consistent with the aptasensor behavior observed for macroelectrode-based sensors, such incubation period at static conditions were chosen for further research.

**Figure 6:**
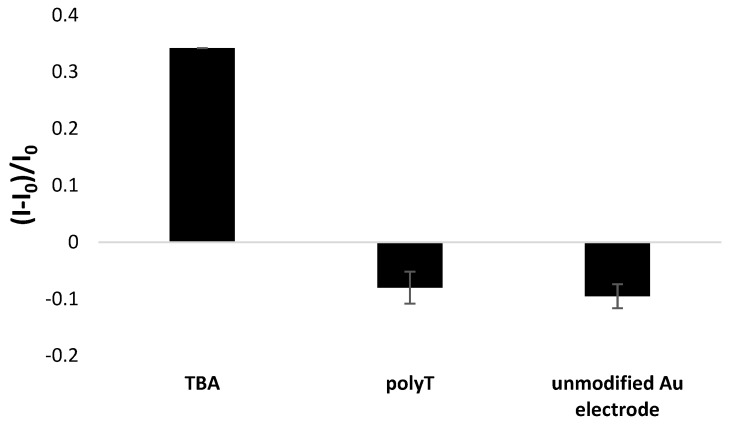
Comparison of TBA/MCH-based and polyT/MCH-based miniaturized gold electrodes response after incubation in 100 nM Pb^2+^ solution containing methylene blue redox indicator (in 50 mM Tris-HCl, pH 4.0).

Importantly, the presence of guanine moieties in the aptamer sequence resulted in a significant increase in aptasensor response when compared with the response of electrode modified with reference ssDNA probe containing solely thymine nucleotides. As seen in Figure 6, a decrease in polyT-modified electrode response was recorded after incubation with 100 nM Pb^2+,^ which confirms the exceptional role of guanine nucleobases and the possibility of the formation of G-quadruplex structure when Pb^2+^ ions are present. Moreover, the application of polyT sequence as a receptor layer led to a response almost equal to that of an unmodified gold electrode. This depicts the necessity of the application of a G-rich aptamer strand to obtain a response of substantial intensity towards lead ions.

**Figure 7:**
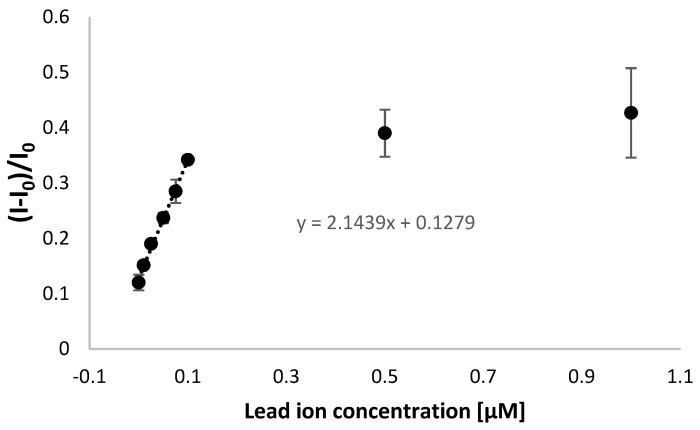
Calibration curve for TBA/MCH-modified electrode for electrochemical detection of lead ions for a wide range of concentrations. The aptasensor response was based on square-wave voltammetry (cathodic scan) measurements in the presence of 50 µM methylene blue redox indicator (in 50 mM Tris-HCl, pH 4.0).

**Figure 8:**
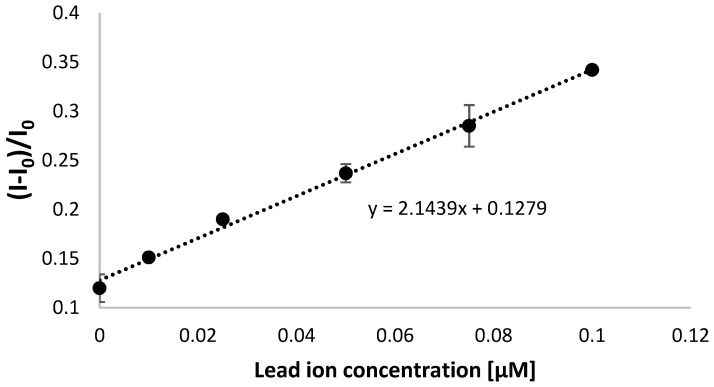
Calibration curve for TBA/MCH-modified electrode for electrochemical detection of lead ions for a narrower range of Pb^2+^ ion concentration reaching 100 nM. The aptasensor response was based on square-wave voltammetry results (cathodic scan) measurements performed in the presence of 50 µM methylene blue redox indicator (in 50 mM Tris-HCl, pH 4.0).

Next, a calibration curve was created based on square-wave voltammetry measurements (cathodic scan), and a linear response was observed between 10 and 100 nM Pb^2+^ ions (Figure 7 and Figure 8). A plot flattening was evident for higher lead ion concentrations, indicating a saturation of the sensing layer with the target analyte. Moreover, the lower limit of detection reached 21 nM, which was calculated using the Equation (2):

(2) $$LOD=\frac{3.3*S_{y}}{S}$$

where *S_y_* is the standard deviation of response for a blank sample (MB solution), and *S* is a slope of a calibration curve.

It should be noted that the obtained range of linear response covers both the concentrations below and above the maximum allowed level set for lead ions, according to the US EPA [43,44]. The miniaturized system also provided the possibility of lead ions detection lower than obtained when applying gold disk macroelectrodes, where the range of linear response was from 0.05 to 1 µM [42]. This implies better conditions in the case of miniaturized systems for distinguishing lead ion concentrations that are harmful to human beings and confirms the improvement of aptamer-based biosensor features for lead ion analysis. It should also be emphasized that a minor increase in aptasensor response for a concentration between 100 nM and 1 µM shows its applicability within the nanomolar range (<100 nM) of lead ions concentration. 

For 50 and 100 nM, the reproducibility studies were conducted, and as can be seen in Table 1, the responses were distinguished with low RSD that did not exceed 5%. Therefore, the high reproducibility of elaborated aptamer-based biosensors was evidenced.

**Figure 9:**
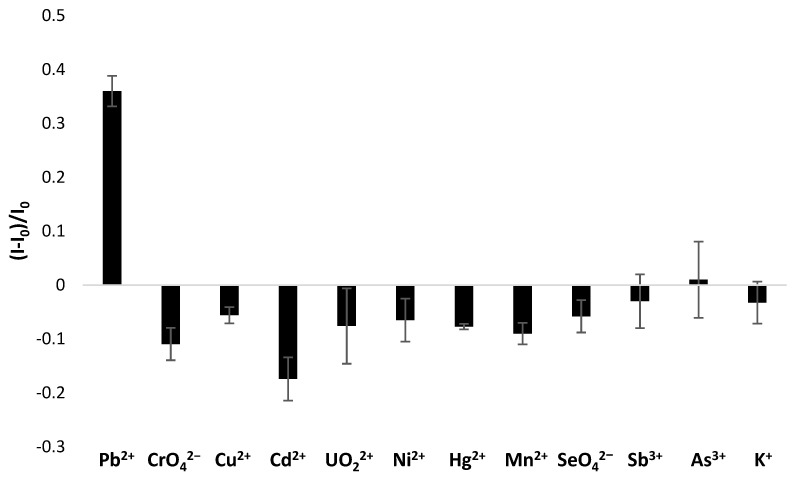
Selectivity studies for TBA/MCH-modified electrode recorded after incubation in 72 nM solution of lead nitrate and interfering heavy metal salts at 72 nM level. The aptasensor response was based on square-wave voltammetry (cathodic scan) measurements in the presence of 50 µM methylene blue redox indicator (in 50 mM Tris-HCl, pH 4.0).

Then, the selectivity of TBA—the TBA-based layer—was studied by comparison of responses at 72 nM concentration, which refers to the maximum acceptable level for lead ions. As seen in Figure 9, in the case of all the analyzed ions, a different mechanism of current response was observed than for the target analyte. Namely, a current decrease was observed after incubation for all the interfering ions. The reason for that could be the exceptional feature of lead ions to stabilize the G-quadruplex structure, causing a sensing layer conformation change and a rearrangement to a more packed form. It could be expected that this would instead cause a repulsion of methylene blue as the overall aptamer layer charge diminished due to Pb^2+^ ions binding to aptamer strands. On the contrary, the formation of G-quadruplexes enabled the exposure of guanine moieties in the outer part of the monolayer. Since there have been several indications of the high affinity of guanines towards methylene blue [46], the accumulation of MB molecules in close vicinity to the electrode surface justifies the increase in the current signal after incubation with lead ions. Such a mechanism did not occur when aptamer-modified electrode was incubated with other tested ions. The current decrease was the most significant in the case of cadmium ions, which can be explained by the possibility of their interaction not only with negatively charged phosphate moieties and oxygen and nitrogen atoms present in nucleotides forming the aptamer strand. Nevertheless, it should be noted that the interaction between interfering ions and the aptamer-based layer was not significant, as the repulsion of methylene blue was minor and led to a smaller current change. Importantly, MB current decrease was observed not only for mono-, di- and trivalent metal cations but also in the case of chromate and selenate anions. Such behavior indicates that the binding of interfering species with the TBA-based layer is substantially weaker than Pb^2+^–TBA interaction. Lastly, the aptasensor response decrease was observed for potassium ions, which are also known to exhibit the ability to stabilize G-quadruplex structures [38]. A distinct tendency of response might be caused most likely by the pH value of solutions used for electrochemical studies. In the case of all experiments, including selectivity studies, mild acidic (pH 4.0) conditions were applied for all tested ions. At higher pH values, lead ions would possibly be complexed by hydroxyl groups, which could affect their affinity towards the aptamer-based layer. On the contrary, potassium ions tend to interact with the TBA sequence in a different manner in mild acidic environments than in conditions like physiological ones, as shown in other studies [38]. Hence, the high selectivity of the TBA/MCH receptor layer towards lead ions was confirmed.

One of the key features of the practical use of developed biosensors is their feasibility for analyzing real samples. As the development of the proposed aptasensor aimed to detect lead ions in water samples, we applied it to analyze tap water samples spiked with lead nitrate at 50 nM concentration. The whole measurement procedure was consistent with the one utilized for studies on laboratory samples, and aptasensor responses were compared after incubation with Pb^2+^ cation solutions prepared in 50 mM Tris-HCl (pH 4.0, containing 50 µM methylene blue redox indicator) and in tap water. In the latter case, a defined amount of lead nitrate was dissolved in tap water to receive 10 mM concentration, which was further diluted in 50 mM Tris-HCl (pH 4.0, containing 50 µM methylene blue redox indicator) to 50 nM concentration. It was expected that the biosensor response for real samples might be affected because of direct contact with a more complex matrix of tap water in comparison to laboratory samples.

**Figure 10:**
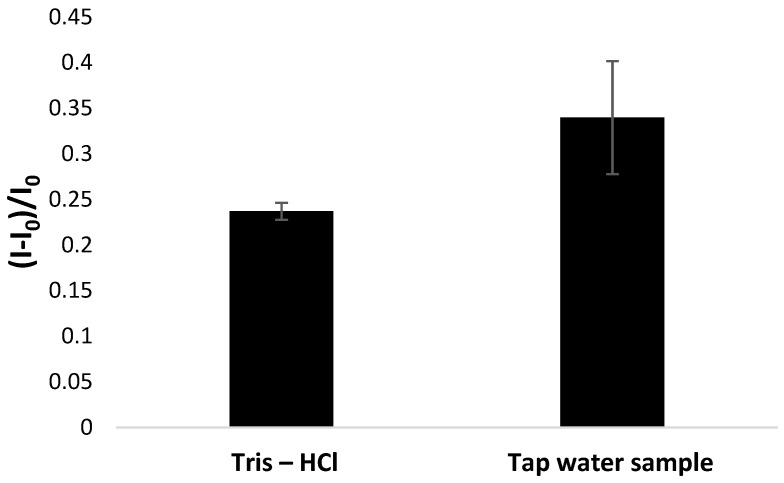
Comparison of TBA/MCH-modified electrode response recorded after incubation in 50 nM lead nitrate solution in 50 mM Tris-HCl (laboratory sample) and in tap water. The aptasensor response was based on square-wave voltammetry measurements (cathodic scan) performed in the presence of 50 µM methylene blue redox indicator (in 50 mM Tris-HCl, pH 4.0).

As can be seen in Figure 10, the current response recorded for a real sample is slightly higher than for the synthetic laboratory sample prepared in 50 mM Tris-HCl solution, pH 4.0. The reason for that is the presence of other components in the water matrix, which influence sensing layer properties and cause its reorganization to a G-quadruplex structure. On the contrary, a partial deterioration of the receptor layer could also happen, which might lead to the introduction of pinholes in the sensing layer. Eventually, this might result in the accumulation of methylene blue molecules at the electrode surface with the prolongation of the incubation period with redox indicator, as it is also present in the lead nitrate solution. Nevertheless, the possibility of applying an elaborated aptasensor to analyze more complex samples was confirmed and proved to be the exact detection mechanism regarding aptamer layer conformation change upon interaction with lead cations.

**Figure 11:**
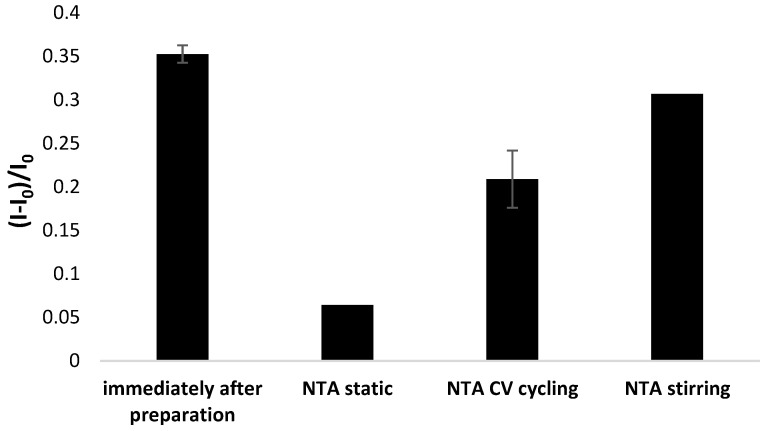
Comparison of aptamer sensing layer regeneration methods referring to using NTA solution (50 mM Tris-HCl). The aptasensor response was based on square-wave voltammetry measurements (cathodic scan) performed in the presence of 50 µM methylene blue redox indicator (in 50 mM Tris-HCl, pH 4.0).

Another issue was verifying the possibility of aptamer-based layer regeneration, and for that purpose, the electrodes were subjected to 1 mM NTA solution and incubated for 5 min at static and stirring conditions and cyclic voltammetry cycling between potentials of −0.6 to 0.2 (10 cycles) at 0.3 V·s^−1^ scan rate was performed. It can be seen in Figure 11 that each method of bioreceptor layer regeneration led to a decrease in response, with the most significant observed for incubation in NTA solution at static conditions. Five minute incubation in 1 mM NTA at stirring conditions allowed the preservation of 87% of the initial aptasensor response, whereas electrochemical scanning caused a 40% drop in response. Therefore, it is possible to regenerate the aptamer-based layer partially. However, it should also be emphasized that screen-printed miniaturized sensors are generally considered single-use transducers.

**Figure 12:**
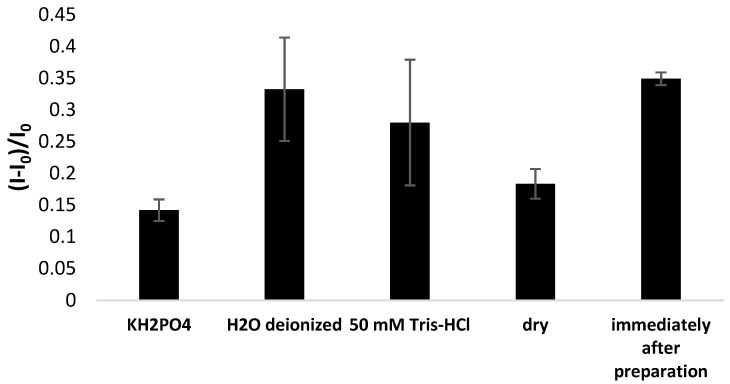
Comparison of aptasensor response immediately after receptor layer preparation and after 72 h storage at 4 °C dry or in specific solutions. The aptasensor response was based on square-wave voltammetry measurements (cathodic scan) performed in the presence of 50 µM methylene blue redox indicator (in 50 mM Tris-HCl, pH 4.0).

The stability of aptamer-based layers is another essential feature concerning the commercial application of the proposed platform and the necessity of transducer storage after fabricating the receptor layers. Hence, the aptasensor response was compared immediately after receptor layer preparation and after 72 h storage at dry conditions in immobilization buffer (1 M KH_2_PO_4_), 50 mM Tris-HCl, and deionized water at 4 °C. It can be seen in Figure 12 that none of the storage methods allowed the initial response to be preserved, and the most significant signal drop was recorded when electrodes were kept in 1 M KH_2_PO_4_ or dry. This indicates a substantial loss of binding properties of the aptamer-based layer and a high likelihood of aptamer strand degradation or detachment from the gold surface. A less pronounced signal decrease was observed when miniaturized electrodes were kept in Tris-HCl (80% of initial response) and deionized water (95% of initial aptasensor response), which suggests that fewer aptamer strands were separated from the gold surface. Therefore, both methods could be considered for application for aptamer-modified electrode storage.

## 4. Conclusions

Herein, we present the results of the application of commercially available miniaturized electrode transducers modified with aptamer strands for electrochemical detection of lead ions. The reason for such an approach is to limit the use of chemicals to fabricate receptor layers and aim for biosensor miniaturization. A thrombin-binding aptamer (TBA) was applied as a receptor element, and methylene blue was used as a current signal source, as in the aptasensor elaborated using gold disk microelectrodes [42]. The conducted studies evidenced a linear response range between 10 and 100 nM, which covers the range below and above the maximum acceptable level of Pb^2+^ in drinking water. Moreover, the high selectivity of the elaborated sensing layer was confirmed for concentration referring to the maximum allowed concentration of lead ions in drinking water (72 nM) [43,44]. Finally, the possibility of analysis of real samples in the form of a tap water sample spiked with lead ions was executed. The more complex matrix can explain the slightly higher response than when the Tris-HCl solution was utilized. The conducted studies confirmed the potential of the use of aptamer-modified miniaturized transducers for lead ions analysis in terms of preservation of binding properties through storage in deionized water or 50 mM Tris-HCl for 72 h and the possibility of partial regeneration of aptamer receptor layer using 1 mM NTA. As seen in Table 2, there are a number of other methods applied for lead ions detection, and some of them are characterized by a wider range of linear response as well as a lower limit of detection. On the contrary, the main advantage of the proposed system is the simplicity of both receptor layer preparation as well as the protocol of electrochemical measurements execution, along with the relatively short time of response. This gives a perspective that the analysis does not require to be executed by a trained operator, so the device could be easily transformed into a generally available tool for monitoring water samples in the areas of threat of contamination with lead wastes or waste collection containing lead. 

Further research could be focused on testing miniaturized transducers with working electrodes made of other metals or carbon materials, analyzing different types of water samples, and elaborating a miniaturized flow system to reduce the analysis time. Future studies could also refer to elaborating a multi-array system for the simultaneous detection of different heavy metal targets, which could give an insight into a broader range of possible contaminants in water samples. Finally, concerning the need for mass production of aptamer-based transducers, this should be proceeded by further studies on the stability of receptor layers so that they could preserve their binding properties upon storage in defined conditions.

## Figures and Tables

**Table 1:** Reproducibility studies of aptamer-based miniaturized sensor for detection of lead ions.

Pb^2+^ Ions Concentration	Biosensor Response (Mean Value ± SD)	RSD
50 nM	0.24 ± 0.009	3.75%
100 nM	0.35 ± 0.01	2.87%

**Table 2:** Comparison of working parameters of aptamer-based biosensors for lead ions detection.

Nature of Pb Detection	Detection Technique	Linear Range of Response	Lower Limit of Detection	Response Time	Reference
L/CuO-CoO-MnO/SiO_2_/IL/CP electrode	SWASV	0.9 nM–230 nM 230 nM–27 µM	0.24 nM	3 min	[68]
Polypropylene hollow-fiber preconcentration	ETAAS	173 pM–2.41 nM	57 pM	40 min	[69]
Immunoassay using the secondary antibody labeled ALP, SA-coated MMPs, and biotin-labeled Cd/Pb-BSA	chemiluminescence	2.4–38 nM	4.8 nM	30 min	[70]
CSAP-AuNPs/SAP/MCH/Au electrode	QCM	5–200 nM	4 nM	45 min	[71]
Disulfide-modified TBA aptamer/MCH/SP Au	SWV	10–100 nM	10 nM	11 min	This work

## Data Availability

Data are contained within the article.
